# Periodontal Regeneration by Allogeneic Transplantation of Adipose Tissue Derived Multi-Lineage Progenitor Stem Cells in vivo

**DOI:** 10.1038/s41598-018-37528-0

**Published:** 2019-01-29

**Authors:** Venkata Suresh Venkataiah, Keisuke Handa, Mary M. Njuguna, Tatsuya Hasegawa, Kentaro Maruyama, Eiji Nemoto, Satoru Yamada, Shunji Sugawara, Lu Lu, Masahide Takedachi, Shinya Murakami, Hanayuki Okura, Akifumi Matsuyama, Masahiro Saito

**Affiliations:** 1 Department of Restorative Dentistry, Division of Operative Dentistry, Tohoku University Graduate School of Dentistry, Sendai, Japan; 2 Department of Oral Biology, Division of Periodontology and Endodontology, Tohoku University Graduate School of Dentistry, Sendai, Japan; 3 Division of Oral Immunology, Department of Oral Biology, Tohoku University Graduate School of Dentistry, Sendai, Japan; 4 Division of Oral Diagnosis, Department of Oral Medicine and Surgery, Tohoku University Graduate School of Dentistry, Sendai, Japan; 5 Department of Periodontology, Osaka University Graduate School of Dentistry, Osaka, Japan; 6 Center for Research Promotion and Support, Fujita Health University, Toyoake, Japan; 7 Department of Regenerative Medicine, Fujita Health University, Graduate School of Medicine, Toyoake, Japan

## Abstract

The ultimate goal of periodontal disease treatment is the reorganization of functional tissue that can regenerate lost periodontal tissue. Regeneration of periodontal tissues is clinically possible by using autogenic transplantation of MSCs. However, autologous MSC transplantation is limited depending on age, systemic disease and tissue quality, thus precluding their clinical application. Therefore, we evaluated the efficacy of allogeneic transplantation of adipose-derived multi-lineage progenitor cells (ADMPC) in a micro-mini pig periodontal defect model. ADMPC were isolated from the greater omentum of micro-mini pigs, and flow cytometry analysis confirmed that the ADMPC expressed MSC markers, including CD44 and CD73. ADMPC exhibited osteogenic, adipogenic and periodontal ligament differentiation capacities in differentiation medium. ADMPC showed high expression of the immune suppressive factors GBP4 and IL1-RA upon treatment with a cytokine cocktail containing interferon-γ, tumor necrosis factor-α and interleukin-6. Allogeneic transplantation of ADMPC in a micro-mini pig periodontal defect model showed significant bone regeneration ability based on bone-morphometric analysis. Moreover, the regeneration ability of ADMPC by allogeneic transplantation was comparable to those of autologous transplantation by histological analysis. These results indicate that ADMPC have immune-modulation capability that can induce periodontal tissue regeneration by allogeneic transplantation.

## Introduction

Periodontitis is an inflammatory disease that causes pathological alterations in tooth-supporting tissues, which can lead to the progressive breakdown of the periodontal tissues including loss of periodontal ligament, cementum and superficial alveolar bone and apical migration of the associated epithelial attachment to form periodontal pocket^1^. Periodontal regeneration requires the formation of new cementum, alveolar bone and a functional periodontal ligament on a previously diseased root surface^2^. Conventional non-surgical or surgical treatments involve reduction or elimination of periodontal pathogens to halt or further control the progression of periodontal disease and results in healing by repair without the formation of new periodontal attachment^2^.

Various therapeutic approaches, including guided tissue regeneration (GTR), platelet-rich plasma (PRP), and enamel matrix derivatives (EMD), have attained success in the regeneration of lost periodontal tissues, but with a relatively high degree of variability^2–4^. Moreover, a variety of recombinant human cytokines have been investigated regarding their ability to stimulate periodontal tissue regeneration. The results of pre-clinical and clinical studies have shown that the application of fibroblast growth factor-2 (FGF-2) facilitates cell proliferation of resident progenitor cells from surrounding bone marrow and PDL and enhances angiogenesis, and bone formation in 2 or 3 wall defects to induce periodontal tissue regeneration^5^. To date, the above-mentioned regenerative procedures have shown that the outcomes of these therapies from both preclinical and clinical studies remain limited to the three bony wall bone defect of periodontal defects, and the results were unpredictable in the case of advanced periodontal defects in which resident progenitor cells are reduced or destroyed^4,6^. Therefore, these therapies should be improved based on stem cell biology, especially those involved in the differentiation of stem cells into PDL, cementum and alveolar bone.

The use of stem cell therapy together with tissue engineering principles to promote periodontal regeneration has attracted increasing attention and has become the focus of research^7^. Mesenchymal stem cells (MSCs) have become an attractive target for use in periodontal regeneration because of their ability to give rise to multiple specialized cell types and their extensive distribution in many adult tissues, including those of dental origin^8^. Hence, autologous transplantation of MSCs in combination with tissue engineering, such as cell sheet technology, has been shown effective for regeneration of the periodontium^9,10^. Adipose-derived multi-lineage progenitor cells (ADMPC) have recently been widely studied as a viable cell source for cell-based regenerative medicine. These cells have shown to have properties similar to other MSCs, with added advantages, such as an easy harvesting procedure and low donor site morbidity^11^. Current evidence suggests that the periodontal microenvironment may induce ADMPC to grow and differentiate into periodontal tissues and that the ADMPC themselves might secrete various factors that stimulate resident progenitor cells^12^. These unique properties appear to make ADMPC an attractive cell source for stem cell-based therapeutic approaches in periodontology. The effect of autologous MSC transplantation has been investigated in clinical trials for periodontal regeneration of healthy patients^13,14^. However, this strategy cannot be used in patients with systemic diseases, such as diabetes, rheumatoid arthritis, systemic lupus erythematosus (SLE), and aged patients in whom the intrinsic properties of MSCs are altered^15^. Hence, there is a need for an allogeneic transplantation approach for patients who experience difficulty with autologous transplantation for periodontal regeneration therapy.

As stated earlier, MSCs, including ADMPC, have immune-modulatory properties; allogeneic MSC transplantation has been extensively investigated for its therapeutic capabilities in a wide variety of diseases, such as brain ischemia, cardiac infarction, osteoarthritis and autoimmune diseases, including SLE and Crohn’s disease^15^. The efficacy and safety of allogeneic MSCs have been investigated in the pre-clinical animal studies for the treatment of periodontal regeneration. The results of these studies have demonstrated a favorable periodontal-regenerative potential of allogeneic MSC^16–21^. Allogeneic ADMPC has also been investigated in various other systemic diseases, such as muscular dystrophy, diabetes, Myocardial Infarction, intervertebral disc degeneration and retinal injury^22^. Because of their immunosuppressive properties and low immunogenicity compared with other cell types, the implantation of allogeneic MSCs may therefore be more reasonable and appropriate^15,22^. ADMPC inhibit the activity of various immune cells, including T, B, natural killer and dendritic cells via cell-cell contact and soluble factors^15^. An inflammatory periodontal disease requires an immune-suppressive treatment strategy using allogeneic ADMPC transplantation, which might contribute as an alternative regenerative therapy to recover functional periodontal tissue.

In this study, we hypothesize that implantation of allogeneic ADMPC will aid in the periodontal regeneration of surgically created periodontal defects. Accordingly, the study aimed to assess the potential of allogeneic ADMPC to form periodontal ligament, cementum and alveolar bone in a micro-mini pig periodontal defect model.

## Results

### Isolation and multi-differentiation capacity of ADMPC

**Figure 1:**
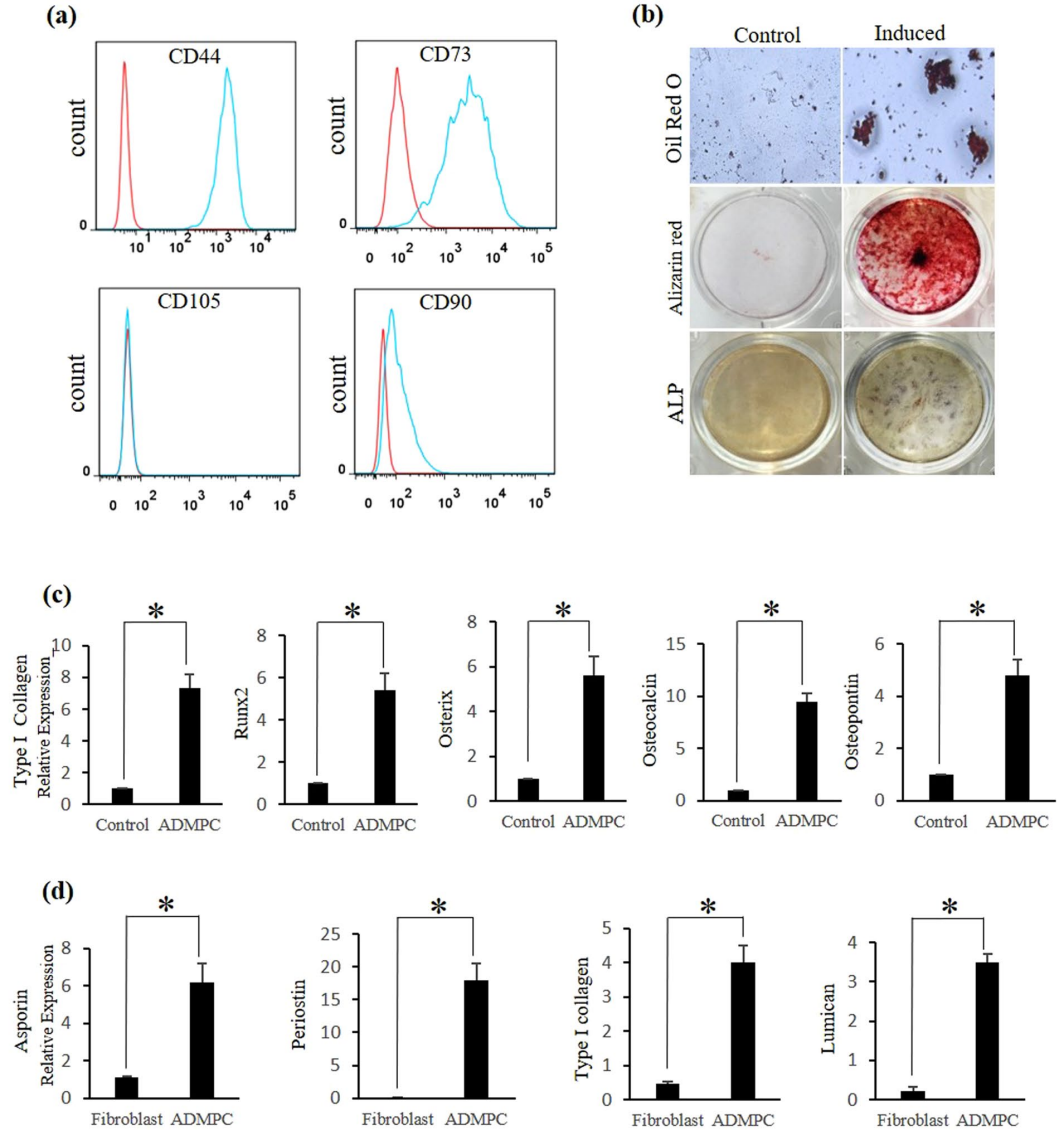
*In-vitro* Characterization of ADMPC. (**a**) Flow cytometry analysis of MSC surface markers on ADMPC. (**b**) Multilineage differentiation ability of ADMPC. Adipogenic and osteogenic differentiation illiustrated in induction medium (upper, middle and lower right panel). Non-induced (control) cultures show a lack of differentiation (upper, middle and lower left panel). (**c**) Osteogenic gene expression of ADMPC cultured in the presence of ODM by real-time PCR. (**d**) Periodontal ligament differentiation ability of ADMPC cultured in the presence of TGFβ by real-time PCR. Data are expressed as the mean ± S.D. *p < 0.05 compared with the control.

Previously, ADMPC isolated from the greater omentum of canines have been shown to possess characteristics similar to bone marrow mesenchymal stem cells (BMMSCs)^23^. Therefore, ADMPC were isolated from the greater omentum of micro-mini pigs, as described in Materials and methods. First, we investigated the MSC phenotype by assessing the expression of MSC markers on these cells using flow cytometry. Immunophenotypic analysis demonstrated that ADMPC expressed moderate to high levels of the MSC surface markers CD44 and CD73 and lacked expression of CD90 and CD105 (Fig. 1a).

In our previous study, ADMPC isolated from beagle dogs demonstrated adipogenic and osteogenic differentiation ability^23^. Therefore, we investigated the adipogenic differentiation ability of ADMPC by the formation of lipid droplets detected by Oil Red O staining after 3 weeks of induction (Fig. 1b, upper right panel). Osteogenic differentiation was demonstrated by the formation of mineralized nodules (Fig. 1b, middle right panel) and blue-violet stained cells (Fig. 1b, lower right panel) by Alizarin red S and alkaline phosphatase staining, respectively. A lack of differentiation was observed in the control groups (Fig. 1b, upper, middle, and lower left panel). To further confirm the osteogenic activity, cells were treated with osteogenic differentiation medium (ODM) for 3 weeks (ADMPC). Real-time PCR analysis was performed to characterize the expression of osteogenic-related genes (Supplementary Table S2, Osteogenic genes) by ADMPC. ADMPC exhibited a clear up-regulation of osteogenic genes such as Type I collagen (7.3 fold), runx2 (5 fold), osterix (5.6 fold), osteocalcin (9.4 fold), osteopontin (4.8 fold) upon treatment with ODM by real-time PCR analysis compared with the control (Fig. 1c). It has been demonstrated that TGFβ enhances the expression of periodontal ligament differentiation markers such as asporin (6.2 fold), periostin (18 fold), type I collagen (4 fold) and laminin, and (3.5 fold) which are dominantly expressed and involved in the development of periodontal ligament^24–26^. Hence, we then investigated the periodontal ligament differentiation ability of ADMPC by assessing the expression of periodontal ligament differentiation markers following TGFβ treatment. Gingival fibroblasts isolated from micro-mini pigs served as a control group. Real-time PCR data showed significantly elevated expression of PDL-related markers by ADMPC compared with gingival fibroblasts (Fig. 1d). These data indicated that ADMPC might have the ability to differentiate into periodontal ligament. Overall, these findings suggest that ADMPC have MSC-like characteristics with the ability to differentiate into multiple lineages.

### Immune-modulation of ADMPC

**Figure 2:**
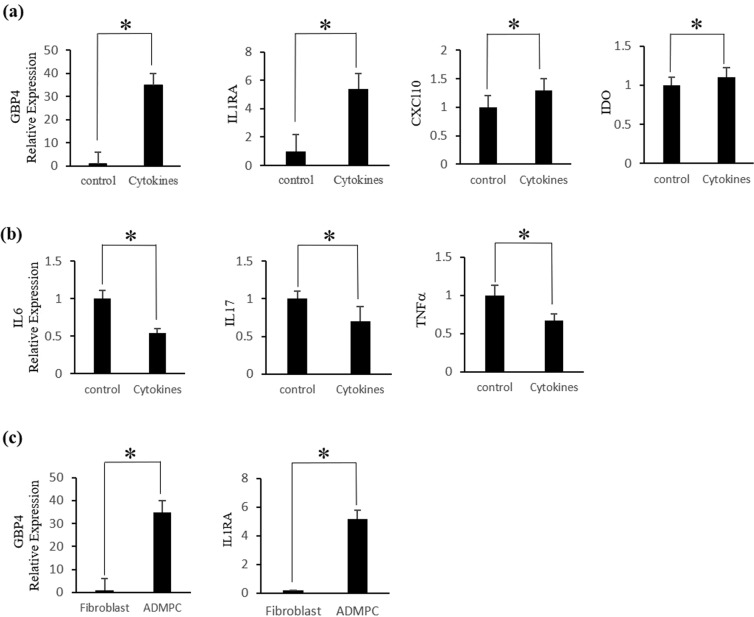
*In-vitro* anti-inflammatory effect of ADMPC. Expression of immunosuppressive and pro-inflammatory-related cytokines by ADMPC treated with cytokine cocktail. Significant up-regulation of the (**a**) immunosuppressive cytokines GBP4, IL1RA, CXCL10, and IDO and down-regulation of the (**b**) pro-inflammatory cytokines IL6, IL17, and TNFα by ADMPC cultured with cytokine cocktail compared with the absence of cytokine treatment. (**c**) GBP4 and IL1RA gene expression of ADMPC compared with gingival fibroblasts. Data are expressed as the mean ± S.D. *p < 0.05 compared with the control.

MSC-dependent immunomodulation is mainly mediated through the regulation of cytokines involved in the inflammatory process^27^. We first investigated whether ADMPC survived in the presence of inflammatory cytokines. The results showed that the morphology of ADMPC did not change with or without cytokine cocktail treatment (Supplementary Fig. S1b). In contrast, gingival fibroblasts showed signs of cell death after four days of cytokine treatment, indicating that the ADMPC could survive in the presence of an inflammatory environment (Supplementary Fig. S1c). We next investigated the expression of immune-suppressive and pro-inflammatory cytokines by ADMPC under inflammatory conditions by real-time PCR. The real-time PCR data showed that ADMPC treated with cytokine cocktail had significantly increased levels of immune-suppressive-related genes, such as GBP4 (33 fold), IL1RA (5 fold) CXCL10 (1.2 fold), and IDO (1.1 fold), compared with the control group (Fig. 2a).

Additionally, the real-time PCR data revealed a significantly small decrease in expression levels of the pro-inflammatory cytokines IL6 8 (0.5 fold), IL17 (0.4 fold) and TNFα (0.4 fold) in ADMPC treated with the cytokine cocktail compared with no cytokine treatment (Fig. 2b). We then examined the expression of GBP4 and IL1RA in ADMPC compared with gingival fibroblasts to confirm whether it was attributed explicitly to ADMPC. The real-time PCR data showed a significant increase in GBP4 and IL1RA expression levels by ADMPC compared to gingival fibroblast (Fig. 2c), indicating increased immune-suppressive effect of ADMPC following cytokine treatment. Overall, these data suggest that ADMPC play an essential role in immunomodulation mediated through the up-regulation of immunosuppressive genes and down-regulation of pro-inflammatory cytokines, thus providing an anti-inflammatory effect.

### Micro-CT (µCT) analysis of new bone regeneration by allogeneic ADMPC

We next established a micro-minipig periodontal defect model to investigate whether allogeneic transplantation of ADMPC could promote periodontal tissue regeneration *in-vivo*. Autologous or allogeneic ADMPC were mixed in fibrin gel and transplanted to the surgically created periodontal defect in the right mandibular third premolars of micro-mini pigs (Fig. S2a,b). All the left mandibular third premolars were transplanted with fibrin gel alone as a control group. At four weeks post-transplantation, the micro-mini pigs were sacrificed and mandibular jaws collected for investigating new bone formation by µCT. After allogeneic transplantation of ADMPC into the periodontal defect, healing was found to progress uneventfully without an intense inflammatory reaction during a four-week observation period. Three-dimensional (3D) reconstruction of µCT images revealed new alveolar bone formation in all the experimental groups. Under the 3D view, the yellow area inside the defect outlined by the arrowhead represents the newly formed alveolar bone. In the coronal, axial and sagittal view of the control group, we could observe the persistence of radiolucency in the defect area (outlined by an arrowhead), suggesting minimal bone formation.

**Figure 3:**
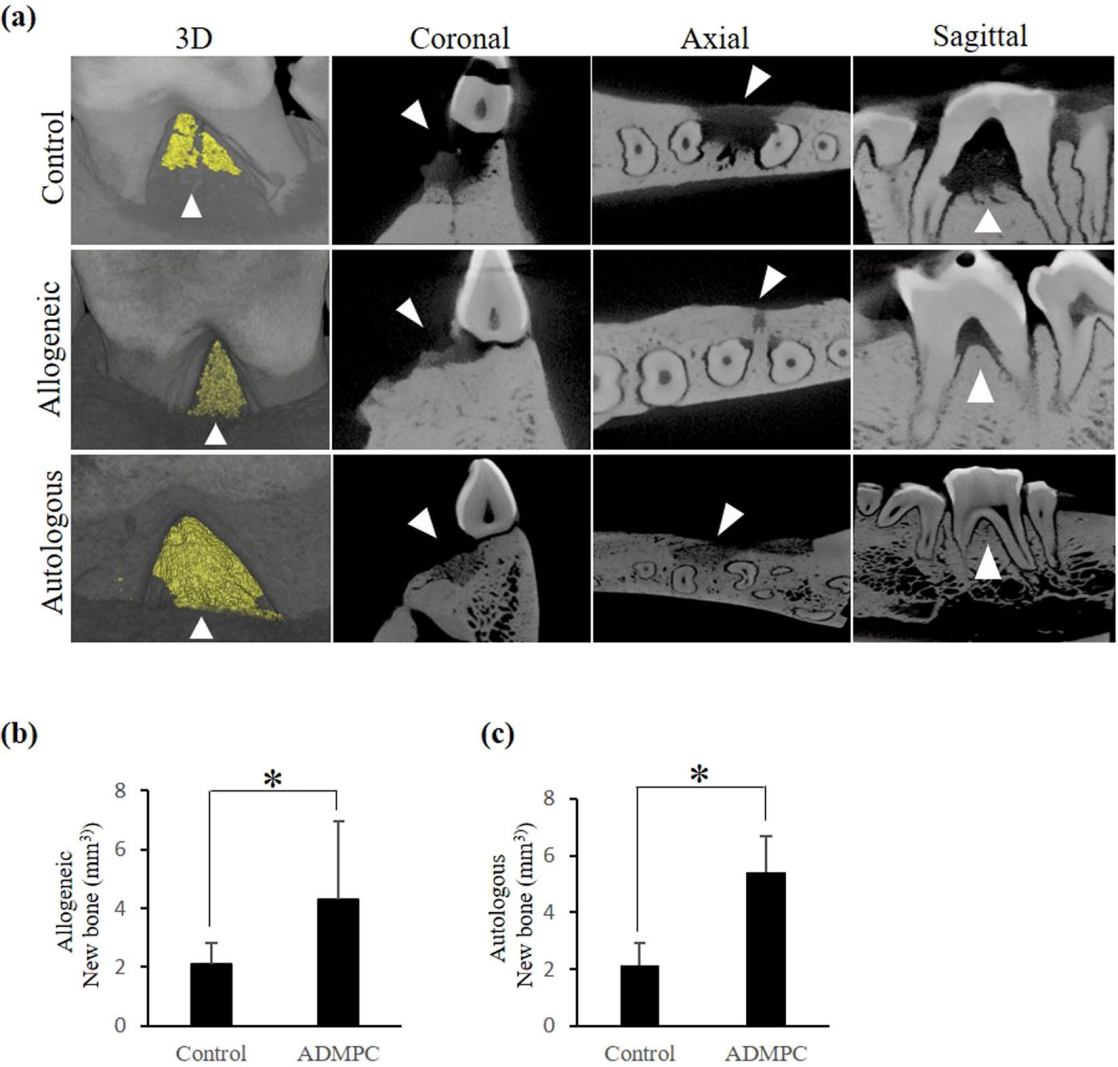
µCT analysis of new bone regeneration by allogeneic ADMPC. (**a**) Three-dimensional reconstruction of µCT images of the furcation defect is shown. The yellow area highlighted by the arrowhead represents new bone formation within the defect area under 3D view. Furcation defect area viewed in the coronal, axial and sagittal views; the persistence of radiolucency (arrowhead) observed in the defect area in the control sample (upper panel) compared with increased radiopacity (arrowhead) in the defect area of allogeneic (middle panel) and autologous (lower panel) ADMPC transplants. Quantification of regenerated alveolar bone from reconstructed three-dimensional µCT images showing that the amount of newly formed bone was significantly higher in (**b**) allogeneic and (**c**) autologous ADMPC transplants compared with the control group. (Data are expressed as the mean ± S.D). *p < 0.05 compared with the control.

In contrast, allogeneic and autologous ADMPC transplanted samples in the coronal, axial and sagittal view showed increasing radiopacity in the defect area (outlined by an arrowhead), suggesting new bone formation (Fig. 3a). Bone volume analysis confirmed that the newly formed alveolar bone ratio in either the allogeneic (4.4 fold) or autologous (5.4 fold) ADMPC transplant group was significantly higher than in the control group (Fig. 3b,c). However, no significant differences were observed concerning the amount of new bone formation in the allogeneic and autologous ADMPC transplanted groups (Fig. 3b,c).

### Histological analysis of periodontal tissue regeneration by allogeneic ADMPC

**Figure 4:**
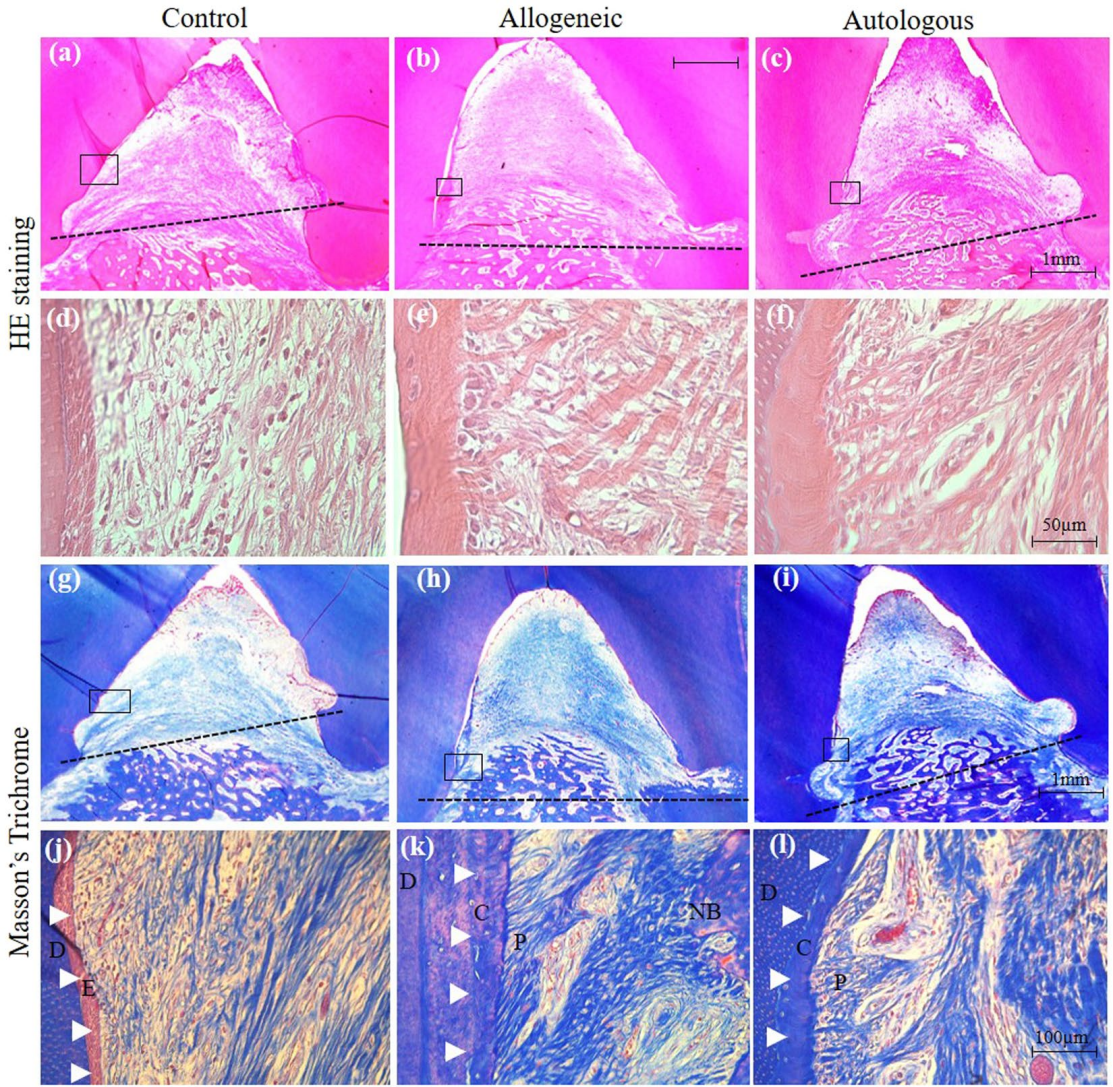
Histological analysis of periodontal regeneration by allogeneic ADMPC. Representative sections of control and experimental samples were examined by hematoxylin-eosin and Masson’s trichrome staining. The dotted line denotes the base of the defect. Minimal new bone regeneration was observed in the (**a**) control sample, whereas a greater amount of new bone regeneration was found in the (**b**) allogeneic and (**c**) autologous ADMPC transplants. (**d**–**f**) Higher magnified sections of the framed regions in (**a**–**c**), indicating immune cell infiltration and collagen fiber formation. (**g**–**i**) Low magnification of MT-stained images of the control and experimental samples observed for cementum and periodontal tissue regeneration from above the notch of the defect area, as indicated by black squares in the photographs. (**j**) High magnification sections of the framed region in (**g**) showing the original cementum area replaced with epithelial tissue (E, arrowhead). (**k**, **l**) High magnification sections of the framed region in (h and i) showing new cementum (C, arrowhead) and periodontal ligament (P) fiber formation. E, epithelial tissue; C, cementum; P, periodontal ligament; NB, New bone.

We then performed a histological analysis to evaluate the periodontal tissue regeneration. The experimental area was sectioned and stained with hematoxylin-eosin (HE) and Masson’s trichrome (MT). HE-stained photomicrographs of the control sample showed minimal bone formation above the base of the defect, which is outlined by a dotted line (Fig. 4a). In contrast, allogeneic and autologous groups showed apparent new bone formation above the base of the defect extending coronally (Fig. 4b,c). Higher magnification of HE-stained images revealed high inflammatory cell infiltration with less collagen fiber formation in the control group (Fig. 4d). In contrast, less inflammatory cell infiltration with high collagen fiber formation was observed in the allogeneic and autologous ADMPC transplant groups (Fig. 4e,f).

We next performed Masson’s trichrome (MT) staining to evaluate cementum and periodontal ligament regeneration. Images of MT staining in the control sample showed no new cementum and periodontal ligament fiber formation. Instead, the original cementum area was replaced with epithelial tissue (E), as outlined by arrowheads (Fig. 4j). In contrast, both the allogeneic and autologous groups demonstrated new cementum (C), as outlined by arrowheads, along with Sharpey’s fibers of the periodontal ligament (P), which were regenerated above the base of the defect and extended coronally (Fig. 4h,i,k,l).

### Immunohistochemistry of leukocyte markers in the defect area

**Figure 5:**
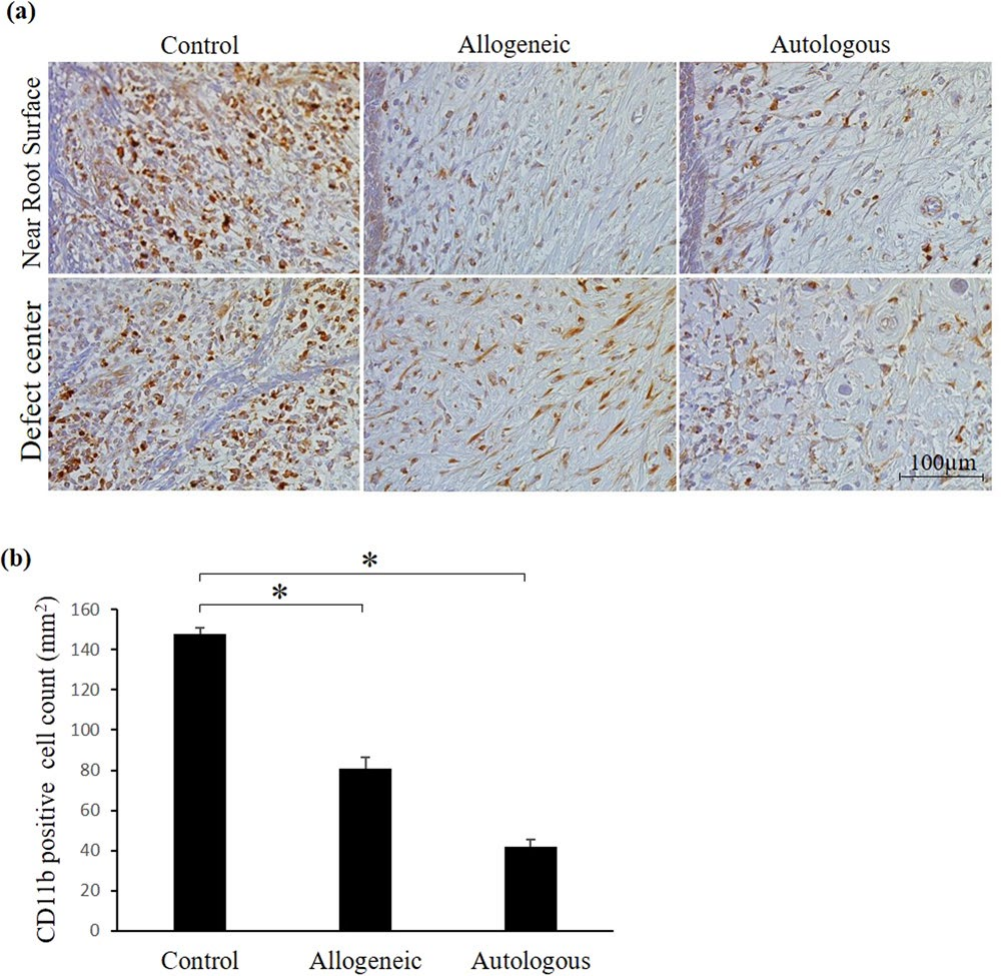
Immunohistochemical analysis of the defect area with anti-CD11b-antibody. Immunohistochemical images showing CD11b stained cells in control, allogeneic and autologous samples. CD11b positive cells were shown as dark brown color. (**a**) Photomicrographs adjacent to cementum (upper panel) and central part of the furcation defect area (lower panel) showed high expression of CD11b-stained cells in the control sample, but weak expression was observed in the allogeneic and autologous ADMPC transplants. (**b**) Quantitative analysis of CD11b-stained cells demonstrated significantly high immune cell infiltration in the control sample compared with the allogeneic and autologous groups. Data are expressed as the mean ± S.D. *p < 0.05 compared with the control.

To investigate the anti-inflammatory effect of ADMPC, we performed immunohistochemical analysis of CD11b as a leukocyte marker. The dark brown stained cells were identified as CD11b positive cells as shown in Fig. 5a. The results indicated that CD11b-positive leukocyte infiltration could not be detected adjacent to the root surface (Fig. 5a, upper panel) and the central part of the furcation defect in the autologous and allogeneic groups (Fig. 5a, lower panel), while infiltration of these cells was observed in the control groups (Fig. 5a, upper and lower panel). Quantitative analysis revealed that the number of CD11b-positive leukocytes was significantly reduced in autologous and allogeneic groups compared with the control (Fig. 5b). These data suggested potential anti-inflammatory role of allogeneic ADMPC mediated through decreased neutrophil infiltration in periodontal defects similar to autologous ADMPC.

### Low immunogenicity of allogeneic ADMPC

Next, we investigated the immunological reaction induced by allogeneic ADMPC transplantation. For this purpose, we evaluated anti-ADMPC antibody induction in the serum collected before and after allogeneic and autologous ADMPC transplantation by FACS analysis. Serum collected before transplantation served as a control group. The results of the FACS analysis demonstrated a small increase in antibody was produced in the post-allogeneic compared to post-autologous ADMPC transplant groups (Fig. S4). However, we did not observe any serious inflammation in both allogeneic and autologous groups. Hence, allogeneic ADMPC could be transplanted for periodontal regeneration therapy without fear of immune rejection.

## Discussion

Regeneration of periodontal tissue requires restoration of cementum, periodontal ligament, and alveolar bone. Cell-based therapy using autologous MSCs has been preferred for periodontal tissue regeneration associated with severe defects. Efficient use of allogeneic MSCs may be an alternative strategy that overcomes the limitations of autologous MSC transplantation procedures for the regeneration of large periodontal defects. Micro-mini pigs have oral and maxillofacial structures similar to humans regarding development, anatomy, pathophysiology and disease occurrence^28^. Thus, in this study, we investigated the feasibility of allogeneic transplantation of ADMPC to regenerate PDL, cementum and alveolar bone in a micro-mini pig periodontal defect model. According to µCT and histological examination, we found that the allogeneic group exhibited significantly higher periodontal tissue regeneration than the control group.

**Figure 6:**
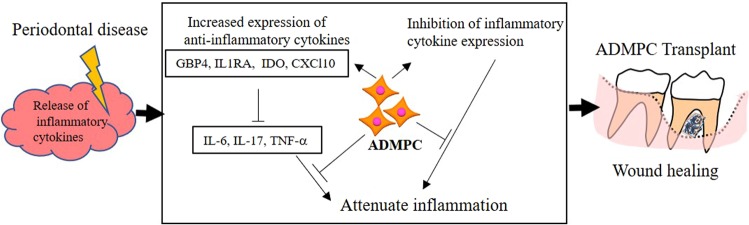
Anti-inflammatory mechanism of ADMPC. In the presence of an inflammatory environment, ADMPC establishes an anti-inflammatory milieu by up-regulating immune-suppressive genes and down-regulating genes encoding pro-inflammatory factors, which in turn down-regulates the inflammatory cytokines produced by the periodontal disease to attenuate inflammation. The immunomodulation properties of ADMPC inhibits progressive inflammation and enhances wound healing.

Furthermore, allogeneic ADMPC also demonstrated an immunosuppressive function similar to MSCs. Our hypothesis regarding the anti-inflammatory mechanisms of ADMPC was explained by an up-regulation of immune-suppressive genes and down-regulation of genes encoding pro-inflammatory factors, which in turn decreased the levels of inflammatory cytokines produced by periodontal disease (Fig. 6). Previous study indicated that allogeneic PDLSCs survived for 8 weeks after transplantation in a dog periodontal defect model^20^. In addition, Yang and colleagues reported GFP labelled allogeneic BMMSCs integrated into newly formed periodontal tissues after transplantation into a rat periodontal defect model indicating direct contribution of transplanted cells to the regenerated periodontal tissues^29^. In the present study, we did not observe serious inflammation or severe tissue destruction after allogenic ADMPC transplantation, suggesting that ADMPC might be survived and integrated in the host tissue certain period of time.

In recent years, evidence has supported the regeneration of periodontal tissues by allogeneic MSC transplantation in periodontal defects using animal models^16,20^. Successful periodontal regeneration has been developed by both allogeneic and autologous MSC transplantation. Periodontal regeneration by autologous MSC transplantation has been well established in our previous laboratory in a dog model, and other researchers have also confirmed the use of various animal models, as explained earlier. Moreover, there are reports that the transplantation of allogeneic MSCs accelerates periodontal regeneration compared with the control group^16,20^. In a previous study, new bone, cementum, and alveolar bone regeneration were recovered to normal levels in both autologous and allogeneic periodontal ligament stem cell (PDLSC) transplantation in a miniature pig periodontal defect model^20^. Similarly, in the present study, the allogeneic group showed higher periodontal regeneration than the control group. These results are consistent with previous findings. The possible mechanism of periodontal regeneration is explained by the immune-modulatory and tissue repair ability of ADMPC. The current consensus based on experimental models indicates that the regeneration of connective tissue is mainly based on the activation of specific signaling pathways involved in the recruitment and mobilization of endogenous MSCs in the defect area^30^.

Tissue destruction in periodontal disease is caused by the intensity and duration of the immune response induced by cytokines^31–34^. Previous reports have indicated that control and modulation of the inflammatory response, mainly the release of cytokines or activation/inhibition in time and spatially controlled manner, might be a possible strategy for periodontal tissue engineering^35^. The imbalance between the production of pro-inflammatory and anti-inflammatory cytokines affects the induction of bone and collagen destruction in periodontal tissue. For instance, activation of TNFα is known to stimulate periodontal bone loss through the inhibition of bone formation. Blocking the effects of these cytokines is critical for preventing periodontal disease, indicating that the inhibition of cytokine activity is essential for preventing periodontal disease. Recent advances have revealed that MSCs suppressed or modulate immune responses by interactions with different types of innate and adaptive immune cells via cell-cell contact and secreted soluble factors^15^. BMMSC-mediated immune suppression of activated T cells has been attributed to the secretion of anti-proliferative soluble factors, such as HGF, TGF-β1, and PGE2^36^. In another study, local injection of BMMSCs inhibited the expression of TNFα, IFNγ, and IL-1β^21^.

Similarly, PDLSC inhibits T-cell proliferation stimulated by mismatched major histocompatibility complex molecules^16^. One of the critical factors involved in the pathogenesis of periodontitis is T cell-mediated immunity^37^. Therefore, utilization of the anti-inflammatory effect of MSCs should be considered as a reasonable alternative approach for the treatment of periodontal diseases. Similar to these studies, our data demonstrated the down-regulation of pro-inflammatory cytokines (TNFα, IL-17, and IL-6) using cytokine cocktail-treated compared with non-cytokine-treated ADMPC, which have been reported to be involved in various inflammatory diseases, including periodontitis. Furthermore, autologous or allogeneic transplantation analysis revealed that the periodontal tissue regeneration occurred through a reduction of neutrophil infiltration. Thus, ADMPC had an anti-inflammatory effect to alter the outcome of the immune cell response by modulating inflammatory cytokine function.

One of the challenges of allogeneic transplantation is to determine the cause of immune rejection and massive cell death of transplanted cells. The possible side effect of allogenic transplantation is chronic inflammation by increasing cell death which is characterized as an increasing immunogenic potential^38^. In the present study, we showed that serum levels of anti-ADMPC antibodies detected after allogenic transplantation, indicating that number of cell death may be increased after allogeneic transplantation. In previous research, allogeneic transplanted MSCs did not cause immune rejection. The referred study investigated immunological reactions by evaluating peripheral blood serum levels of CRP, CD30, IL-10 and IFNγ and found no significant differences between autogenic and allogeneic groups in a dog periodontal defect model^20^. In addition, preclinical and clinical trials have reported that allogeneic hematopoietic stem cell transplantation can alleviate GVHD and lead to improved outcomes^15^.

Moreover, MSCs express low or modest levels of MHC class I molecules and lack expression of MHC class II and co-stimulatory molecules, leading to low immunogenicity and thus avoiding immune responses in recipients^15^. The detailed mechanism of the immune response following allogeneic transplantation must be further investigated. The current data suggest that neither rejection nor tissue damage occurs in the allogeneic transplantation model. This finding is consistent with previous results showing no marked differences between the periodontal-regenerative capacity and immune rejection response following allogeneic MSC transplantation^16,20^. Thus, present investigation complements existing evidence for the ability of allogeneic ADMPC is beneficial compared to MSC transplantation or other existing therapy. There are studies shown that ADMPC have phenotype and genotype are similar to other MSCs^11^. ADMPC can be obtained with less invasive methods, minimal donor site morbidity and high cell number than other MSCs^11^. Therefore, the use of ADMPC provides a significant added advantage compared to other MSCs.

Mechanistically, the ADMPC produced high immunosuppressive factors (GBP4 and IL-1RA) in co-cultures with the cytokine cocktail. Guanylate-binding proteins (GBPs) are interferon-stimulated factors that are involved in defense against cellular pathogens and inflammation. These proteins have been mainly established as reliable markers of IFNγ activated cells in various diseases, including colorectal carcinoma (CRC) and inflammatory bowel disease (IBD)^39^. Interleukin-1 (IL-1) is one of the pivotal cytokines in the initiation and progression of rheumatoid arthritis and various other inflammatory diseases, and IL-1 receptor antagonist (IL-1Ra) has been shown to block its effects^18,40^. In addition, the expression levels of the immune-suppressive factors IDO and CXCL10 were higher compared with the control group after treatment with the cytokine cocktail. The increased immunosuppressive effect of ADMPC was mainly dependent on the increased expression of GBP4 and IL1-RA, as well as, to some extent, IDO and CXCL10. Taken together, ADMPC establishes an anti-inflammatory milieu by increasing the expression of anti-inflammatory cytokines and down-regulating the expression of pro-inflammatory cytokines, which might be responsible for immunomodulation and thus capable of preventing progressive inflammation.

Autologous MSC transplantation has been shown to have a favorable effect on periodontal tissue formation, as displayed by the positive impact of cell-based approaches on the new bone, cementum and PDL formation^12,23,41–43^. Although many clinical trials have demonstrated  the efficacy of autologous MSC transplantation, their clinical application is limited by age restrictions and systemic disease, which increase the difficulty of isolating MSCs^15,44^. The present study demonstrated that the allogeneic ADMPC therapeutic approach could regenerate periodontal tissues in larger periodontal defects in a pre-clinical micro-mini pig model. An understanding of the detailed mechanisms responsible for the periodontal tissue regeneration and immune-modulation will require further investigations.

## Materials and Methods

### Isolation and characterization of ADMPC

This study was reviewed and approved by the animal care and use committee of the Tohoku University Graduate school of Dentistry (2014DnA-36-3). All the animal experimental procedures performed based on approved guidelines of Regulations for Animal Experiments and Related Activities at Tohoku University. Eight healthy micro-mini pigs (Fuji Micra, Japan), aged 24–30 months and weighing 25–35 kg, were used and analyzed in the experiments. The greater omentum was resected from each micro-mini pig following general anesthesia. Briefly, the resected omentum was minced and digested at 37 °C for 90 minutes with 0.25% collagenase (Wako) in phosphate-buffered saline and centrifuged as described previously^23^. The cell pellet was suspended in 2 ml of PBS and filtered through a 70 µm cell strainer. Density gradient centrifugation excluded the red blood cells with Histopaque (Sigma, UK), and the remaining cells were cultured in MF medium (Toyobo) until confluent. The cells were then washed with PBS and treated with 0.02% ethylenediaminetetraacetic acid (EDTA) (Nacalai tesque) for 2 minutes. Floating cells were collected and seeded on fibronectin (Wako)-coated dish in MF medium and maintained as ADMPC.

### Flow Cytometry activated cell sorting (FACS) analysis of ADMPC

To analyze the MSC surface marker expression on ADMPC, flow cytometry analysis using specific fluorochrome-conjugated monoclonal antibodies was utilized. 1 × 10^5^ cells were washed with flow cytometry buffer consisting of 1% bovine serum albumin (BSA) and 0.1% sodium azide in PBS and subsequently labeled with monoclonal antibodies (Supplementary Table S1) for 30 mins. Isotype-identical antibodies served as controls for each antigen. Cells were gated on FSC and SSC to eliminate debris and cell aggregates using a flow cytometer (BD LSR Fortessa, BD Biosciences). The results were analyzed using FlowJo software.

### Osteogenic Differentiation of ADMPC

To induce osteogenic differentiation, ADMPC were cultured in 48 well plate until confluent and were then treated with osteogenic differentiation medium (ODM) consisting of MF medium supplemented with, 10 mM b-glycerophosphate, 50 µg/ml ascorbic acid, 10 nM dexamethasone, and 200 ng/ml recombinant human bone morphogenetic protein (rhBMP)-2 (R&D systems), as described previously^45^. ADMPC without ODM served as a control group. Extracellular matrix calcification was determined on fixed ADMPC stained with 1% alizarin red solution (Wako) for five mins. ALP activity was detected by staining with ALP staining solution consisting of 0.1 mg/ml naphthol AS-MX phosphate (Sigma Aldrich), 0.6 mg/ml Fast Blue B salt (Sigma Aldrich), 2 mmol/L MgCl_2_ (Wako), 0.5% N,N dimethylformamide (Wako) and Tris-HCl (pH 8.5) at room temperature as described previously^46^. Photographs were obtained to compare the intensity of staining between the control and ODM cultures.

### RNA and qPCR analysis for osteogenic-related gene expression

To evaluate the osteogenic gene expression in ADMPC, 1 × 10^5^ cells were cultured for 3 weeks with or without ODM. Total RNA from cultured cells was isolated using ISOGEN II (Nippon Gene, Tokyo, Japan) according to the manufacturer’s protocol. cDNA was synthesized from 1 µg of total RNA in a 20 µL reaction containing 10X reaction buffer, 1 mmol/L of dinitrophenol phosphate (dNTP) mixture, 1 U/ L RNase inhibitor, 0.25 U/ L reverse transcriptase, and 0.125 mol/L random hexamers (Takara, Tokyo, Japan). The cDNA was then amplified, and specific gene expression using swine-specific primers (Supplementary Table S2, Osteogenic genes) was quantified using a real-time PCR apparatus (Bio-Rad CFX Connect, Applied Biosystems) for 40 cycles following the reaction profile: 95 °C for 3 mins, 55 °C for 30 sec, 65 °C for 5 sec. The expression of the tested osteogenic genes was calculated using the 2^−ΔCT^ method compared with housekeeping gene GAPDH.

### Adipogenic Differentiation of ADMPC

To induce adipogenic differentiation, ADMPC were cultured were cultured in 48 well plate until confluent and were treated with adipogenic differentiation medium (ADM) consisting of MF medium supplemented with, 0.5 µM dexamethasone, 1 µM 3-isobutyl-1-methylxanthine, 200 µM indomethacin and 1 µg/ml insulin. ADMPC without ADM used as a control group. After 3 weeks, the cells were fixed with 10% formalin for 20 mins and stained with fresh Oil Red O solution (20 mg/ml) for 15 mins as described previously^23^. Differentiation was examined microscopically (Axiocam 506 Mono, Zeiss) by observation of lipid-laden vacuoles.

### Periodontal ligament Differentiation of ADMPC

To investigate periodontal ligament gene expression of ADMPC, cells were seeded on a 60 mm culture dish until confluent. The cells were then serum starved for 24 hours. Subsequently, the cells were maintained in MF medium supplemented with 10 ng/ml of TGFβ (Wako) for 1 week, as described previously^47^. Gingival fibroblasts obtained from micro-minipigs were used as a control group. Real-time PCR was performed to analyze the expression of periodontal marker genes (Supplementary Table S2, Periodontal ligament markers).

### *In-vitro* anti-inflammatory effect of ADMPC

To evaluate the immunomodulatory function of ADMPC, 5 × 10^5^ cells were seeded in a 6-well plate. The cells were serum starved for 24 hours. The cells were then treated with MF medium supplemented with a cytokine cocktail (50 ng/ml IFNγ, 20 ng/ml TNFα and 10 ng/ml IL-6) for 7 days, as described previously^27^. Real-time PCR analysis was performed to assess the expression of immune-suppressive related genes and pro-inflammatory cytokines (Supplementary Table S2, Immunosuppressive and Pro-inflammatory markers). Next, we investigated highly expressed immune-modulatory factors in ADMPC compared with gingival fibroblasts to determine whether this effect could be attributed specifically to ADMPC.

### Furcation Defect Model and ADMPC Transplantation

All surgical procedures were performed under general anesthesia (sevoflurane) and local anesthesia solution with 2% lidocaine. Furcation defects were prepared in mandibular premolars of micro-mini pigs (Fig. S2a,b). The defects were created using sterile carbide burs in a low-speed handpiece under continuous sterile saline irrigation. Reference notches were placed on each root to indicate the basal level of the defect. The dimensions of the defects were verified with a periodontal probe and filled with silicone impression paste (Tokuyama Dental Corp, Tokyo, Japan) to induce inflammation. Four minipigs each were randomly assigned to autologous and allogeneic transplantation. The right side of the defects was transplanted with autologous or allogeneic ADMPC-fibrin gel complex and the left side with fibrin gel (CSL Behring) alone. Serum was collected from all experimental micro-minipigs before and 4 weeks after transplantation to evaluate the immunological response to allogeneic transplantation.

### Bone morphometric analysis

Micro-mini pigs were euthanized after four weeks of transplantation, and mandibles were dissected. Bone regeneration was examined by micro-computed tomography (µCT) scanning. Three-dimensional µCT images were analyzed and quantified using image analysis software (TRI/3D-BON, Ratoc System Engineering, and Tokyo, Japan).

### Histological analysis

Following the µCT analysis, the tissues were decalcified in 50% formic acid and 20% citric acid for 4 months. The samples were then dehydrated through a graded series of ethanol, cleared with xylene and embedded in paraffin. Serial sections (5 µm) were cut in the mesiodistal plane throughout the buccal-lingual extension of the teeth. The sections were deparaffinized and stained with hematoxylin-eosin (HE) and Masson’s trichrome to evaluate newly formed alveolar bone, cementum, and periodontal ligament under a light microscope (Leica, DM6000B).

### Immunohistochemical analysis

The formalin-fixed, paraffin-embedded specimens were cut into 4-μm-thick sections and placed on glue-coated glass slides for immunohistochemistry. Briefly, the samples were deparaffinized in xylene and hydrated in a graded alcohol series and distilled water. Endogenous peroxidase activity was blocked with 3% hydrogen peroxidase for 10 min. Antigen retrieval was performed in citrate buffer (pH 9.0) by autoclaving (Tomy SX-500 High-Pressure Steam Sterilizer, Tomy Seiko Co., Ltd., Tokyo, Japan) and heating at 121 °C for 5 min. The samples were then incubated for 30 min at room temperature (RT) in a blocking solution with 1% rabbit serum (Nichirei Bioscience, Tokyo, Japan). Then, the anti-CD11b (1:100, EPR1244, Abcam, Cambridge, United Kingdom) monoclonal antibody reaction was performed for 16 h at 4 °C. A secondary antibody reaction was performed using an Envision + System-HRP labeled polymer anti-mouse antibody for 30 min at RT, and DAB (3, 3 -diaminobenzidine) was used to visualize the binding of the first antibody. Then, the sections were examined using a Leica DM6000B (Leica, Wetzlar, Germany) fitted with a digital video camera (Leica MC 170HD, Leica). Quantification of CD11b positive cells were counted in ten randomly selected high power (20x) fields in the defect area manually.

### Statistical analysis

The results were analyzed and collected using GraphPad Prism 6 software (La Jolla, CA, USA). Samples were tested in triplicate for each *in-vitro* test. Means and standard deviations were calculated and statistical significance of the difference between groups was examined using one-way analysis of variance (ANOVA) followed by Dunnett’s multiple comparison test. Test results were considered significant for probability values of p < 0.05.

### Immunological response to Allogeneic Transplantation

To investigate antibody formation against allogeneic ADMPC transplantation, FACS analysis was performed using fluorochrome-conjugated rabbit anti-pig antibody, as described previously^48^. The peripheral blood was collected from experimental pigs at two specific time points: before (0 weeks) and after (4 weeks) transplantation. The blood samples were centrifuged for 10 mins to separate serum from other blood components. The transplanted ADMPC and serum were incubated for 1 hour and washed with PBS. To evaluate anti-allogeneic ADMPC antibody, FITC-coupled rabbit anti-pig antibody (Cell Signaling) was used. Serum collected before transplantation served as a control group. An isotype control, rabbit IgG antibody was substituted for the primary antibody. Cell fluorescence was determined by flow cytometry (BD LSR Fortessa, BD Biosciences).

## Supplementary information

Supplementary Information
