# Supplementary material for: Periodontal Regeneration by Allogeneic Transplantation of Adipose Tissue Derived Multi-Lineage Progenitor Stem Cells in vivo
Source: Sci Rep. 2019 Jan 29;9:921. doi: 10.1038/s41598-018-37528-0 (PMC6351614; doi:10.1038/s41598-018-37528-0)
Supplement: Supplementary file 1 — Supplementary Information [file 41598_2018_37528_MOESM1_ESM.docx]

**Supplementary information**

**Periodontal Regeneration by Allogeneic Transplantation of Adipose Tissue Derived Multi-Lineage Progenitor Stem Cells in vivo**

V. VENKATA Suresh^1^, Keisuke HANDA^1^, Mary M. NJUGUNA^1^, Tatsuya HASEGAWA^1^, Kentaro MARUYAMA^2^, Eiji NEMOTO^2^, Satoru YAMADA^2^, Shunji SUGAWARA^3^,
Lu LU^3,4^, Masahide TAKEDACHI^5^, Shinya MURAKAMI^5^, Hanayuki OKURA^6^, Akifumi MATSUYAMA^7^ and Masahiro SAITO^1^ *

^1^Department of Restorative Dentistry, Division of Operative Dentistry, Tohoku University Graduate School of Dentistry

^2^Department of Oral Biology, Division of Periodontology and Endodontology, Tohoku University Graduate School of Dentistry

^3^Division of Oral Immunology, Department of Oral Biology, Tohoku University Graduate School of Dentistry, Sendai, Japan

^4^Division of Oral Diagnosis, Department of Oral Medicine and Surgery, Tohoku University Graduate School of Dentistry, Sendai, Japan

^5^Department of Periodontology, Osaka University Graduate School of Dentistry

^6^Center for Research Promotion and Support, Fujita Health University

^7^Department of Regenerative Medicine, Fujita Health University, Graduate School of Medicine

Corresponding author

Masahiro Saito D.D.S, Ph.D.
Professor
Department of Restorative Dentistry 
Division of Operative Dentistry
Tohoku University Graduate School of　Dentistry
4-1 Seiryo-machi, Aoba-ku, Sendai, Japan,980-8575 
Tel: +81-22-717-8340
Fax: +81-22-717-8344
E-mail:mssaito@dent.tohoku.ac.jp

**Supplementary Methods**

**Morphology and Growth kinetics of ADMPC under inflammatory condition**

To establish appropriate culture conditions for ADMPC growth in an inflammatory environment, a cell proliferation assay was performed. For this purpose, ADMPC (1x 10^3^ cells/well) were seeded in a 96-well plate under the following conditions: 5% FCS with or without the cytokine cocktail (50 ng/ml IFNγ, 20 ng/ml TNFα and 10 ng/ml IL-6 ) or 1% FCS with or without the cytokine cocktail. The culture medium was replaced every 2 days, and the cell proliferation assay was performed at 1, 4 and 7 days using the Cell Counting Kit-8 (Dojindo, CK04-11). On the day of cell counting, cells were stained with 10 µl of the CCK-8 solution in each well and incubated at 37°C, 5% CO2 for 4 hours. The optical density absorbance was determined at 450 nm using a Versa Max TM microplate reader.

Morphological changes of ADMPC under inflammatory environment was investigated by culturing ADMPC with and without cytokine cocktail for 7 days. Gingival fibroblast was used as a control group. Photographs of cell pictures were taken by inverted microscope (Zeiss Microscope Axio Vert. A1) at 1, 4 and 7 days to assess the morphological changes.

**Furcation Defect Model and ADMPC Transplantation**

Mandibular third premolars on both sides of minipigs were used for the experiments. A total of 8 micro-mini pigs (16 periodontal defects) were used. The root surfaces were carefully scaled and root planed with rotary instruments and a surgical curette to remove all the cementum. Four weeks after furcation defect preparation and before transplantation, clinical assessment and probing depth measurements were performed, and the defects were thoroughly cleaned by scaling and root planed. Next, 7.5x10^6^ ADMPC were mixed with fibrin gel (CSL Behring) to prepare the ADMPC fibrin gel complex. The right side of the defect was transplanted with allogeneic or autologous ADMPC-fibrin gel complex and the left side with fibrin gel alone (control) in all experimental micro-mini pigs. The flaps were then repositioned and sutured with 4-0 resorbable silk suture (Ethicon). Four weeks after transplantation, the micro- mini pigs were sacrificed, and the lower jaw was collected for micro-computed tomography (µCT) analysis to evaluate new bone regeneration.

**Supplementary Fig. S1**


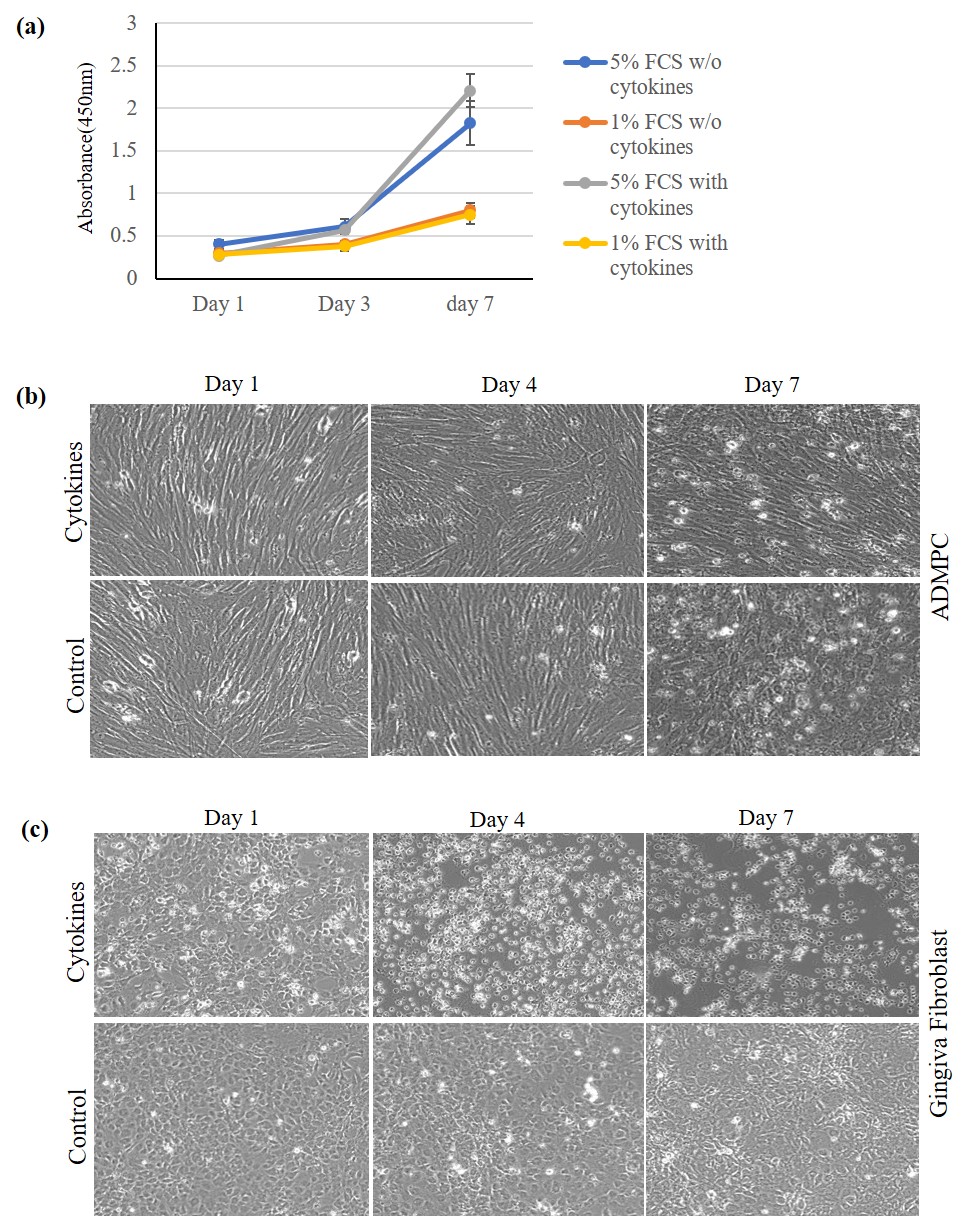


**Supplementary Fig. S2**


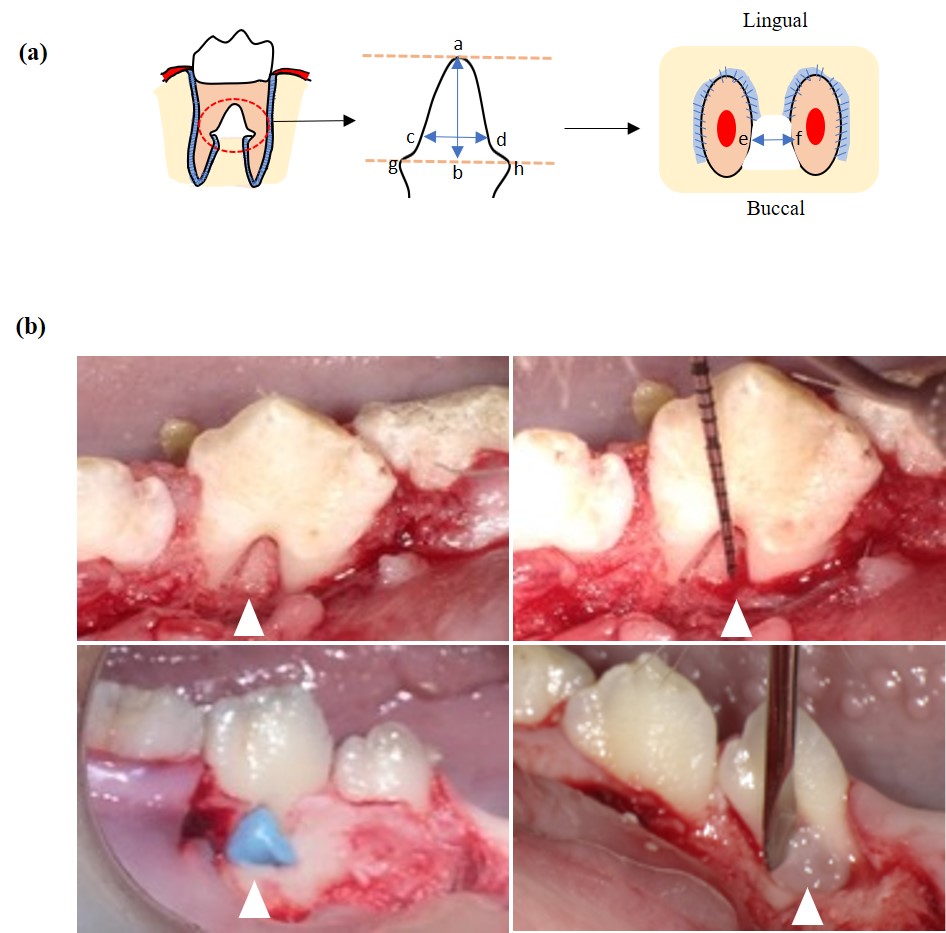


**Supplementary Fig. S3**

**
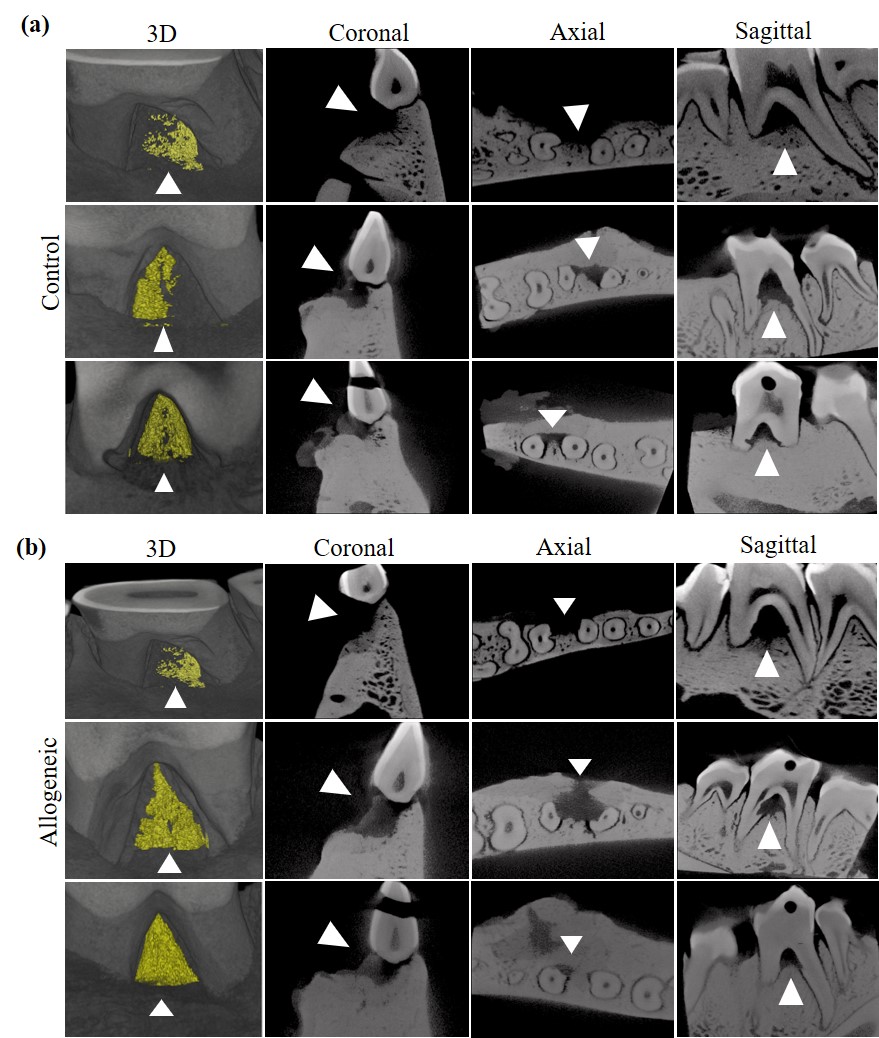
**

**Supplementary Fig. S3**


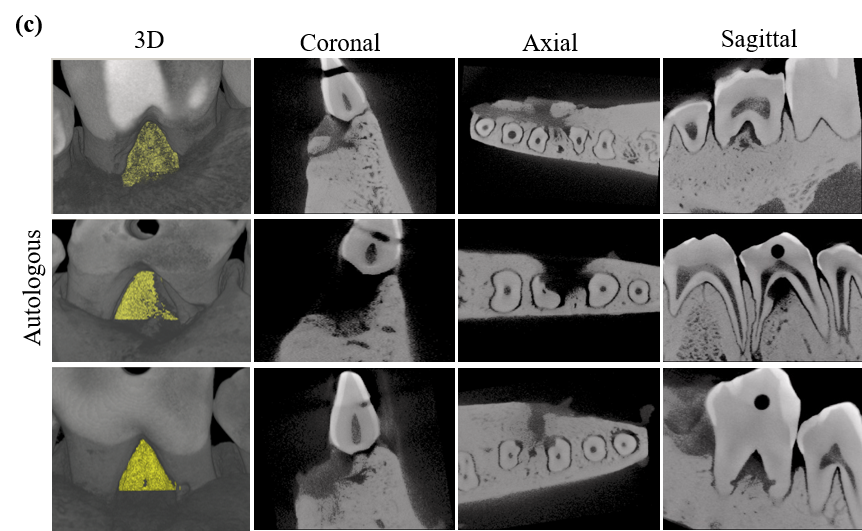


**Supplementary Fig. S4**


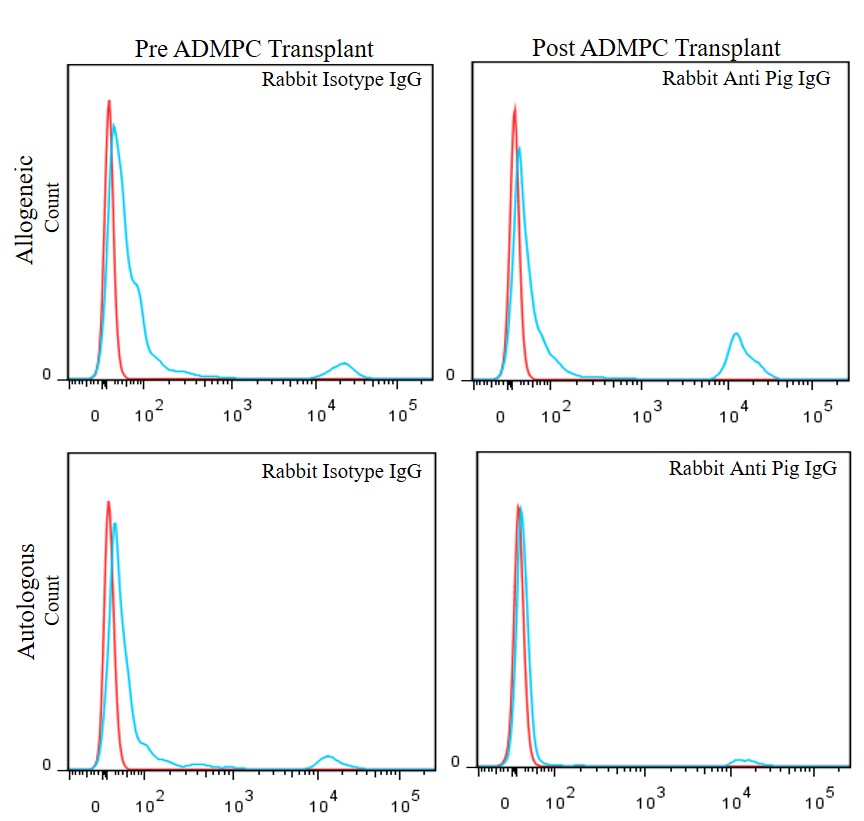


**Supplementary Figure Legends**

**Supplementary Fig. S1**

**ADMPC Growth and Morphology in an inflammatory environment**

(a)The data illustrated that 5% FCS with the cytokine cocktail increased cell proliferation, which was obvious on day 7, compared to 5% FCS without cytokine treatment. However, no significant difference was observed in the cell growth of ADMPC cultured with 1 % FCS treated with or without the cytokine cocktail at 1, 3 and seven days. Overall, ADMPC viability in the presence of 1% FCS with cytokine cocktail was relatively similar to that with 1 % FCS without cytokine cocktail treatment. Hence, 1% FCS with cytokine cocktail was chosen as a proper concentration to mimic the inflammatory environment in vitro for further experiments.
(b, c) Morphological alterations of ADMPC and gingival fibroblasts following cytokine cocktail treatment. (b) ADMPC treated with cytokines retained a normal cell morphology with a spindle shape feature similar to those without cytokine treatment (control). (c) Gingival fibroblasts treated with cytokines exhibited obvious morphological changes with a rounded shape decreased intercellular spaces and ultimately underwent cell death, which was characterized by cell body atrophy and cytoplasm shrinkage.

**Supplementary Fig. S2**

**Establishment of the periodontal defect and ADMPC transplantation**

(a) Schematic drawing of the established furcation defect shown in the dotted circle, a-b: 7 mm height; c-d: 5 mm width; e-f: 5 mm deep; g-h: reference notches. (b) Pre-clinical images of the established furcation defect and ADMPC-fibrin gel complex transplantation in the mandibular third premolar in the micro-mini pig. Furcation defect preparation (upper left) and measurement with the periodontal probe for standardization (upper right). Impression paste placed in the defect area to induce inflammation (lower left). Fibrin gel complex with and without transplanted ADMPC in the defect area (lower right).

**Supplementary Fig. S3**

**In-vivo new bone volume regeneration by µCT analysis.**

Three-dimensional reconstruction of µCT images demonstrating new bone regeneration in the defect area along with coronal, sagittal and axial views of the (a) allogeneic, (b) autologous and (c) control (c) groups are shown (n=4).

**Supplementary Fig. S4**

**Sensitization test for allogeneic ADMPC transplantation**

Anti-ADMPC antibody production was investigated in the serum of pre and post-allogeneic or autologous ADMPC transplantation. Rabbit isotype IgG was used as a control.

**Supplementary Table S1**

Monoclonal antibodies used to study antigenic phenotype on cultured ADMPC

| **Monoclonal Antibodies** | **CD/specificity** | **Fluorochrome** | **Supplier** |
| --- | --- | --- | --- |
| Anti-mouse/human | CD44 | FITC | Biolegend |
| Rat IgG2B, k isotype control | IgG2b k | FITC | Biolegend |
| Anti-CD 105 | CD 105 | FITC | Ancell |
| Anti-human CD90(Thy1) | CS 90 | FITC | Biolegend |
| Anti-m/pCD73 (IgG) | CD73 | PE | R&D systems |
| Normal Sheep IgG | IgG | PE | Santa Cruz technology |
| Anti-Sheep IgG(H+L) | IgG(H+L) | PE | R&D systems |

**Supplementary Table S2**

|  | **Forward Sequence** | **Reverse Sequence** |
| --- | --- | --- |
| **Osteogenic Genes** | | |
| Type I collagen | CCCAGCCGCAAAGAGTCTAC | CAGGTGACTGGTGGGATGTC |
| RUNX2 | GCGGTGCAAACTTTCTCCAG | AATGCCTCTGTTGGTCTCGG |
| Osterix | GCTGTGAAACCTCAAGTCCTATGG | TCCAAGCCAATGTCCTCCTC |
| Osteocalcin | CATAGCCTAGACCTCGCAGC | ATGGGGACCTTACACTTGCC |
| Osteopontin | TGATAGCCTTCTGCCTCTGG | TCGTCCACATCGTCTGTTTG |
| GAPDH | GGACCAGGTTGTGTCCTGTG | CCACCACCCTGTTGCTGTAG |
| **Periodontal Ligament Markers** | | |
| Asporin | TGATGCTGAAGGACATGGAA | AATGTTGCTTGGGACAGAGG |
| Periostin | TTAGCTTGCCTGTTGGCTCT | AGTCGGATTCGTTTCCACTG |
| Type I collagen | CCCAGCCGCAAAGAGTCTAC | CAGGTGACTGGTGGGATGTC |
| Lumican | CTCTTGGTGACAATCGGCAG | GCACCATTGGCAGCTTTTCA |
| **Immune Suppressive Genes** | | |
| GBP4 | GCATCTGGATGTGGTGTGTC | GGTTGATGGTGTTCATGCTG |
| IL1RA | TGTGCCTGTCCTGTGTCAAG | CAGGCTGGTCTGCCTCTAGT |
| CXCL10 | AGAGTCGAAGGCCATCAAGA | GGGATGATGAACCATCTGCT |
| IDO | TAGGCTTTGCTCTGCCAAAT | ACGTACGCCATGGTGATGTA |
| **Pro-inflammatory Cytokines** | | |
| IL-6 | GACAAAGCCACCACCCCTAA | TGGACGGCATCAATCTCAGG |
| IL17 | CTCGTGAAGGCGGGAATCAT | TGGAGAGTCCATGGTGAGGT |
| TNFα | GCCCTTCCACCAACGTTTTC | TCCCAGGTAGATGGGTTCGT |

Nucleotide sequence of primers used for real time PCR. The primers were designed for swine clone sequence.
